# The effect for hyperuricemia inpatient of uric acid overproduction type or in combination with topiroxostat on the pharmacokinetics, pharmacodynamics and safety of dotinurad, a selective urate reabsorption inhibitor

**DOI:** 10.1007/s10157-019-01817-3

**Published:** 2019-11-16

**Authors:** Daisuke Okui, Tomomitsu Sasaki, Masahiko Fushimi, Tetsuo Ohashi

**Affiliations:** Medical R&D Division, Development Department, Fuji Yakuhin Co., Ltd., 4-383, Sakuragi-cho, Omiya-ku, Saitama, Saitama 330-9508 Japan

**Keywords:** Hyperuricemia, Selective urate reabsorption inhibitor, URAT1 inhibitor, Dotinurad, Uric acid overproduction type, Topiroxostat

## Abstract

**Background:**

Dotinurad, a novel selective urate reabsorption inhibitor (SURI), increases urinary uric acid excretion. The aim of this study is to examine the pharmacokinetics, pharmacodynamics, and safety of dotinurad according to the type of hyperuricemia, with or without concomitant use of xanthine oxidase inhibitor, in uric acid “overproduction type” patients.

**Methods:**

This open-label clinical pharmacology study was conducted in a hospital. Dotinurad 1 mg was administered for 7 days to hyperuricemic patients with uric acid “overproduction type” (overproduction group, *n* = 6; and combination group, *n* = 6) and uric acid “underexcretion type” (underexcretion group, *n* = 6). In the combination group, topiroxostat 80 mg was used concomitantly.

**Results:**

No significant differences were observed in pharmacokinetics and safety between overproduction group and underexcretion group, and the percent change in serum uric acid level and the amount of urinary uric acid excretion after administration were comparable. In “overproduction type” patients of combination group, the percent change in serum uric acid level significantly increased and the amount of urinary uric acid excretion significantly decreased compared to those of overproduction group. No clinically meaningful differences were observed in safety between the overproduction group and the combination group.

**Conclusion:**

In inpatients, differences in hyperuricemic type did not significantly influence the pharmacokinetics, pharmacodynamics, and safety of dotinurad. Moreover, in “overproduction type”, the coadministration of dotinurad and topiroxostat had an add-on serum uric acid lowering effect and suppressed urinary uric acid excretion.

**Trial registration:**

ClinicalTrials.gov Identifier: NCT02837198.

**Electronic supplementary material:**

The online version of this article (10.1007/s10157-019-01817-3) contains supplementary material, which is available to authorized users.

## Introduction

Persistent hyperuricemia can lead to urate crystal deposition diseases such as gouty arthritis, urinary tract calculi and renal impairment [1]. Properly maintaining serum uric acid levels is important for the prevention of these diseases and can further lead to the protection of renal function and the reduction of cardiovascular disease risk [2, 3].

Improving lifestyle is important in the treatment of hyperuricemia. It is however difficult to relieve the symptoms of patients with repeated gouty arthritis or with gouty tophus by merely improving lifestyle; hyperuricemic treatment should thus be conducted to maintain serum uric acid levels ≤ 6.0 mg/dL [1]. Hyperuricemia is mainly classified into three types according to cause: uric acid overproduction “overproduction type”; decrease in uric acid excretion “underexcretion type”; and the combination of both “combined type”. In Japan, prevalence of each is estimated to be 10%, 60%, and 30%, respectively [1]. Japanese guidelines for the management of hyperuricemia and gout recommend treating “underexcretion type” with uricosuric drugs (benzbromarone, probenecid) and “overproduction type” with xanthine oxidase inhibitors (XOIs) (allopurinol, febuxostat). For “overproduction type”, the administration of uricosuric drugs is considered inappropriate because that increases the risk of urinary calculi [4].

Dotinurad is a novel selective urate reabsorption inhibitor (SURI), that selectively inhibits urate transporter 1 (URAT 1). In non-clinical study, it was confirmed that the inhibition of uric acid reabsorption by administrating dotinurad results in the promotion of uric acid excretion into urine and the reduction of serum uric acid levels [5]. Since dotinurad promotes the excretion of uric acid into the urine, according to the Japanese management guidelines it would be primarily administered to “underexcretion type” patients. However, in the clinical setting, improvement in serum uric acid levels or relief of symptoms such as gouty attacks during pharmacotherapy may affect the patient’s lifestyle, such as eating habits, and lead to a change in the hyperuricemic type [6]. This is probably because changes in body purine intake from diet, as well as changes in the total body pool resulting from treatment, affect changes in urinary urate excretion, one of the indices used for hyperuricemia classification. In addition, the possibility of dotinurad being administered to “overproduction type” patients cannot be ruled out, given that the fundamental principle of the Japanese management guidelines may sometimes not be observed in selecting drugs to avoid side effects [4]. Therefore, we considered it useful and necessary to examine the pharmacokinetics (PK), pharmacodynamics (PD), and safety of dotinurad in “overproduction type” patients.

Japanese management guidelines also report that concomitant therapy with XOIs and uricosuric drugs is effective [7, 8], and these drugs can be used concomitantly in clinical settings. Dotinurad and an XOI may thus be used together clinically. Therefore, we considered the PK, PD, and safety investigations of the concomitant use of dotinurad and an XOI (topiroxostat) should be required.

## Methods

### Study design

This Japanese study was conducted as an open-label, 7-day, multiple dose clinical pharmacology study in hospitalized Japanese patients. Classification of hyperuricemia was conducted twice, once at screening and once on the day before initial administration (day − 1).

**Fig. 1:**
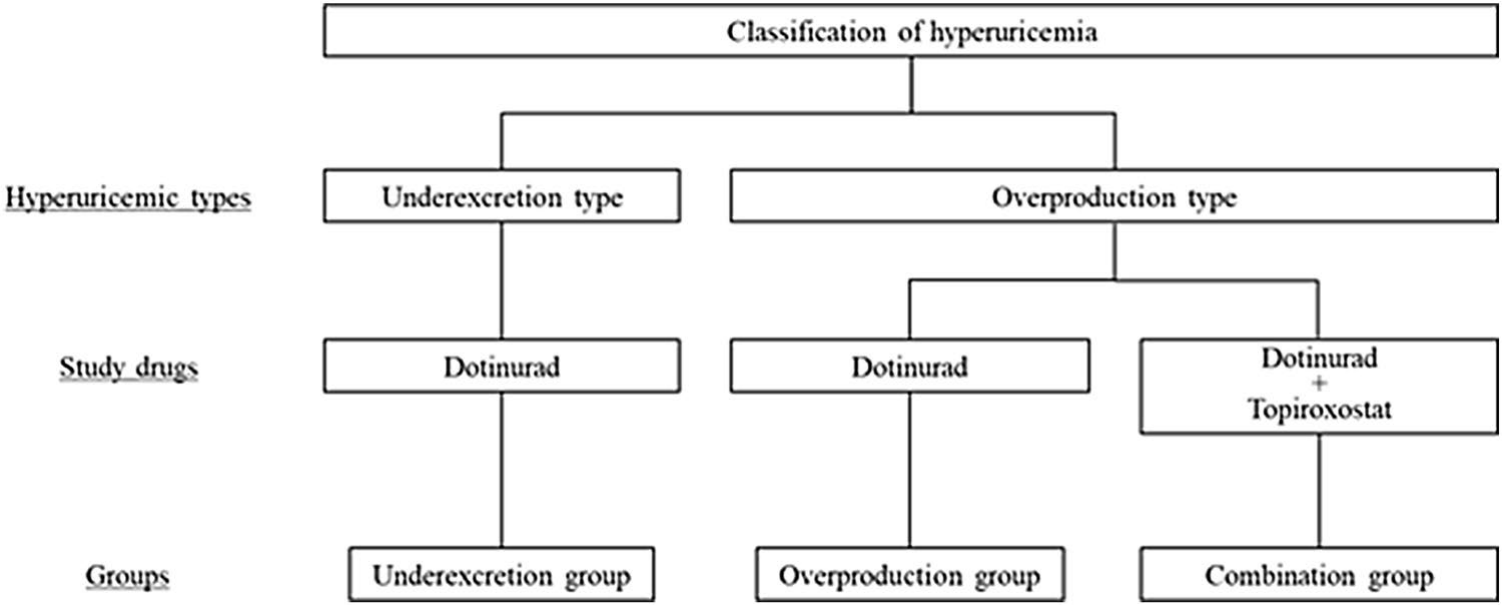
Group configuration

Patients who were deemed eligible for participation in this study at the screening examination were hospitalized and underwent hyperuricemia classification on day − 1. “Underexcretion type” patients were assigned to the underexcretion group. On the other hand, “overproduction type” patients were assigned to either the overproduction group or combination group. The patients in the overproduction and underexcretion groups were treated with dotinurad 1 mg; those in the combination group were treated with dotinurad 1 mg and topiroxostat 80 mg (Fig. 1). Patients received study drugs for 7 days (days 1–7) once daily after breakfast. The health status of the patients who completed study drug administration was assessed for 2 days (days 8–9) after the final administration. During days 1–9, colchicine and citrate were concomitantly administered to prevent gouty attacks and urinary calculi.

### Protocol amendment

In the initial protocol of this study, the patients were hospitalized for 4 days before initial administration and had low purine intake based on the Japanese management guidelines. However, during the hospitalization period until the initial administration, the meals and living environment were very different from the usual lifestyle pattern of these patients. Therefore, the hyperuricemic type on day − 1 changed from that reported at screening, and none of “overproduction type” patients could participate in this study. We thus modified the protocol (i.e., the hospitalization period before initial administration was reduced to 1 day). Although six “underexcretion type” patients had already completed administration using the initial protocol, we decided that the study should be conducted again in all groups using the modified protocol because all groups required evaluation under the same conditions, based on the study aims. “Underexcretion type” patients who had completed administration before the protocol amendment were not used in the analyses of this study because the protocol differed from that of the other patients, and their results are shown as the ‘reference group’ in a supplement.

### Inclusion and exclusion criteria

The main inclusion criteria of this study are described below.

Patients with serum uric acid levels ≥ 7.0 mg/dL at screening examination and on day − 1; patients who were classified as “overproduction type” or “underexcretion type”; and male patients aged at least 20 years.

The main exclusion criteria are described below.

Patients with a history of surgery involving the digestive organs (e.g., the gastrointestinal tract, liver, gallbladder, bile duct, pancreas) or kidney, excluding surgery not affecting drug absorption (e.g. cecum and hemorrhoid surgery); patients who changed the dosage of drugs or used drugs that may affect serum uric acid levels in the 14 days between the screening examination and initial administration; patients with a history of allergy (including hypersensitivity) to topiroxostat, the combination of potassium citrate and sodium citrate hydrate, or colchicine; patients with a history of urinary calculus or patients with a urinary calculus observed on abdominal ultrasonography or simple abdominal X-ray examinations at the screening examination; patients with an eGFR < 30 mL/min/1.73 m^2^ at the screening examination; patients who consumed alcohol after the day prior to admission; patients who consumed grapefruit or substances containing grapefruit within 7 days before the day of admission; patients who consumed *Hypericum perforatum* (St. John wort) or substances containing *Hypericum perforatum* within the 4 weeks before admission; patients who had previously participated in clinical trials of dotinurad and received administration of the study drug; patients otherwise judged to be unsuitable for participation in this study by the principal investigator.

### Blood and urine sample collection

Plasma samples were collected 25 times for PK measurements. Blood samples were obtained at the following times: on days 1 and 7, before administration and 0.5, 1, 2, 3, 4, 6, 8, and 12 h after administration; on days 2–6, before administration; on days 8 and 9, 24 and 48 h after the final administration. Serum samples were collected 28 times for PD measurements. Blood samples were obtained at the following times: on days 1, 4, and 7, before administration and 1, 2, 4, 8, and 12 h after administration; on days 2, 3, 5, and 6, before administration and 12 h after administration; on days 8 and 9, 24 and 48 h after the final administration. Urine samples were collected at the following times: on day − 1, between 24 h before initial administration and just before initial administration; on days 1–7, between 0 and 24 h after administration each day; on day 8, between 24 and 48 h after the final administration.

### Classification of hyperuricemia

Hyperuricemia was classified into the following four types based on uric acid measurement in the 60-min urine collection at screening and on day − 1 [4]: (1) uric acid overproduction type, urinary excretion of uric acid (E_UA_) > 0.51 mg/kg/h and uric acid clearance (C_UA_) ≥ 7.3 mL/min/1.73 m^2^; (2) uric acid underexcretion type, E_UA_ < 0.48 or C_UA_ < 7.3; (3) combined type, E_UA_ > 0.51 and C_UA_ < 7.3; and (4) normal type, 0.48 ≤ E_UA_ ≤ 0.51 and C_UA_ ≥ 7.3. Patients classified as combined type and normal type were excluded from this study.

### Analytical methods

The dotinurad concentration in the plasma was measured using liquid chromatography-tandem mass spectrometry (LC–MS/MS) at the research institute, Fuji Yakuhin Co., Ltd.

The Agilent 1100 series HPLC system (Agilent Technologies, USA) was used for the liquid chromatography; API3000 (AB SCIEX, USA) was used for the mass spectrometry; and Inertsil ODS-3 (150 mm × 2.1 mm, 3 μm, GL Sciences Inc., Japan) was used for the analysis column. Dotinurad concentrations were measured using 5 mmol/L ammonium acetate solution (pH 4.0)/methanol (50:50) in the mobile phase.

The lower limit of quantification was 1 ng/mL for measurement of dotinurad concentration in plasma.

Uric acid concentrations in serum and urine were measured by the enzyme method at the clinical trial institution.

### Pharmacokinetic analyses

The PK parameters of dotinurad were calculated by the non-compartmental model using WinNonlin V6.4. The main PK parameters used for the PK evaluation were as follows: maximum plasma concentration (*C*_max_), time to reach the peak plasma concentration (*T*_max_), elimination half-life (*T*_1/2_), area under the plasma concentration–time curve from time zero to infinity (AUC_0-inf_), elimination rate constant (kel), distribution volume/fraction of dose absorbed (Vd/F), total clearance/fraction of dose absorbed (CL_tot_/F), and mean residence time from zero to infinity (MRT_0-inf_). The AUC was calculated using the linear trapezoidal method by selecting linear trapezoidal linear interpolation as the WinNonlin calculation method.

### Pharmacodynamic analyses

The PD parameters were as follows: delta maximum effective concentration (ΔEC_max_), delta area under the serum concentration–time curve (ΔAUEC_0–24_), maximum reduction rate, amount of uric acid excreted in urine from time zero to 24 h (Ae_0–24_), renal clearance from time zero to 24 h (CL_R0–24_), and fractional uric acid excretion from time zero to 24 h (FE_0–24_). ΔAUEC_0–24_ was calculated from AUEC_–24-0_ and AUEC_0–24_. AUEC_–24–0_ was calculated by extrapolating the serum uric acid level before initial administration on the day of initial administration to 24 h before initial administration. FE_0–24_ was calculated by correcting CL_R0–24_ using creatinine clearance.

### Safety evaluations

Adverse events (AEs), adverse drug reactions (ADRs), and safety assessments were conducted by principle investigators based on clinical laboratory test values (including Ccr), vital signs, and 12-lead electrocardiography. AEs were classified according to the system organ class and preferred term (MedDRA/J ver. 20.0; Japanese Maintenance Organization, Tokyo, Japan) and were evaluated in terms of their potential causality with the study drug, severity, and seriousness. AEs for which a causal relationship with the study drug could not be ruled out by the investigator were considered to be ADRs.

### Statistical analyses

The number of subjects per group was determined to be six as minimum required one to evaluate pharmacokinetics and pharmacodynamics.

Statistical analyses were performed using SAS system version 9.2 (SAS Institute, Cary, NC, USA). For comparisons between groups, the Dunnett multiple comparison test was used with a two-sided significance level of 5%. Dunnett multiple comparison was performed for the underexcretion group and the combination group by setting the overproduction group as the standard. Paired t-tests were performed to compare measurements before and after administration.

Differences in hyperuricemic types were compared in the overproduction group and underexcretion group. Conversely, a comparison between “overproduction type” patients with/without coadministration of topiroxostat was conducted in the overproduction group and the combination group, where dotinurad monotherapy was administered in the overproduction group and concomitant therapy with dotinurad and topiroxostat was administered in the combination group.

## Results

### Subjects

**Fig. 2:**
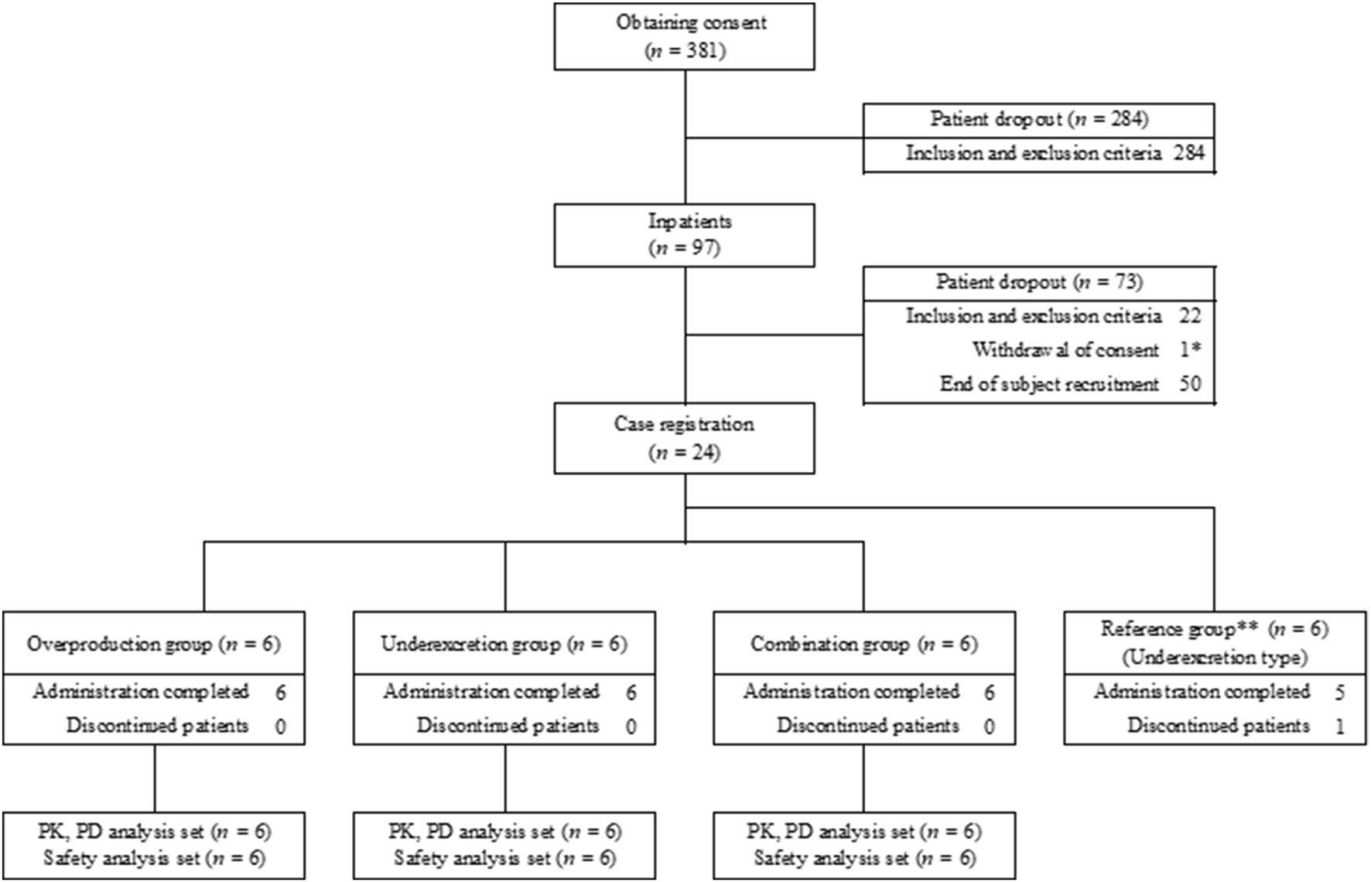
Flow diagram of study protocol. *Classification of hyperuricemia was not conducted on day – 1. **Results in the reference group are shown in the supplement

Informed consent for this study was obtained from 381 patients. After screening, 284 patients were excluded and 97 patients were hospitalized. Of the 97 patients, 73 were excluded. The main reason for exclusion was the hyperuricemic type at screening or day − 1. Finally, study drugs were administered to 24 hyperuricemic patients (six patients in each group). Of the 24 treated patients, one patient (reference group) discontinued the study (Fig. 2). The discontinued patient met the prespecified discontinuation criteria (the urinary uric acid excretion increased by ≥ 15% from day 1 of administration). No differences in characteristics of patients were observed among all groups (Table 1).

**Table 1:** Summary of the baseline characteristics

Category	Treatment groups		
	Overproduction (*n* = 6)	Underexcretion (*n* = 6)	Combination (*n* = 6)
Age (year)	63.0 ± 9.7	52.8 ± 8.2	50.3 ± 6.0
Height (cm)	170.85 ± 4.73	164.35 ± 2.54	169.32 ± 3.68
Weight (kg)	72.62 ± 9.85	69.40 ± 6.54	77.67 ± 7.62
BMI (kg/m^2^)	24.92 ± 3.68	25.75 ± 2.98	27.05 ± 2.01
eGFR (mL/min/1.73 m^2^)	74.0 ± 14.1	73.7 ± 8.4	80.8 ± 4.4
Serum uric acid level (mg/dL)	7.68 ± 0.47	8.30 ± 0.93	8.32 ± 0.50

Values are presented as the mean ± SD

eGFR (mL/min/1.73 m^2^) = 194 × Serum creatinine^−1.094^ × Age^−0.287^

*BMI* body mass index

### Pharmacokinetics

#### PK of dotinurad in the overproduction and underexcretion groups

**Fig. 3:**
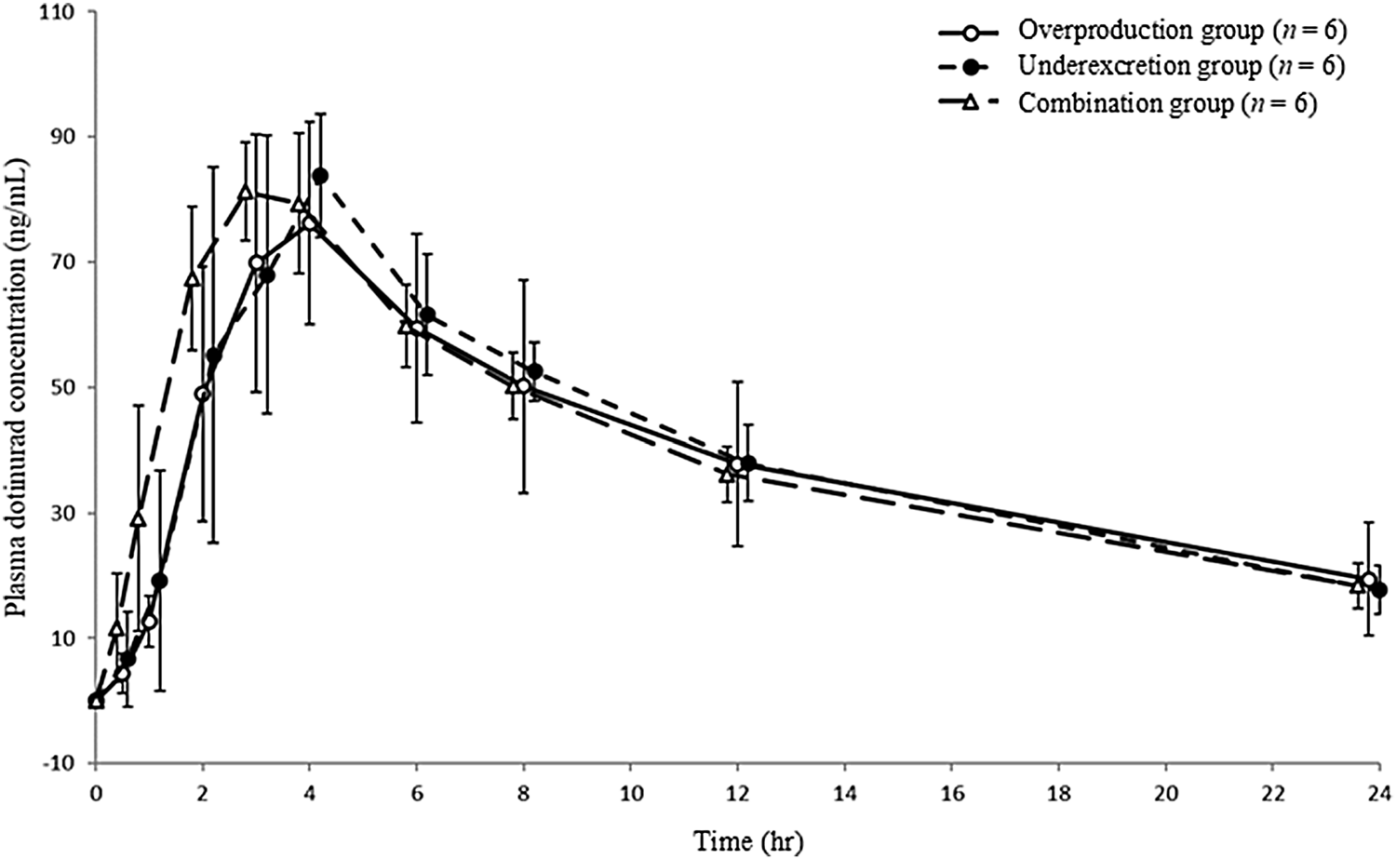
Change in plasma concentration of dotinurad on day 1 in the overproduction, underexcretion, and combination groups. Error bars indicates standard deviation

In the overproduction and underexcretion groups, the mean plasma dotinurad concentrations at all timepoints on day 1 were comparable (Fig. 3). In these groups, all PK parameters (*C*_max_, *T*_max_, *T*_1/2_, AUC_0-inf_, CL_tot_/*F*, kel, Vd/*F*, and MRT_0-inf_) were comparable (Table 2).

**Table 2:** PK parameters of dotinurad in plasma and urine on days 1 and 7 in the overproduction, underexcretion, and combination groups

Parameters	Treatment groups		
	Overproduction (*n* = 6)	Underexcretion (*n* = 6)	Combination (*n* = 6)
*C*_max_ (ng/mL)			
Day 1	78.68 ± 14.92	83.80 ± 9.77	83.28 ± 10.22
Day 7	102.90 ± 21.43	101.20 ± 18.28	108.33 ± 15.49
*T*_max_ (h)			
Day 1	3.50 ± 0.55	4.00 ± 0.00	3.17 ± 0.41
Day 7	3.67 ± 0.52	3.17 ± 0.98	3.67 ± 0.52
*T*_1/2_ (h)			
Day 1	11.67 ± 1.92	10.24 ± 1.32	11.37 ± 2.23
Day 7	11.49 ± 1.49	10.44 ± 0.66	10.83 ± 0.96
AUC_0-inf_ (ng h/mL)			
Day 1	1277.29 ± 490.68	1224.51 ± 197.78	1274.93 ± 155.18
Day 7	1688.49 ± 633.70	1561.90 ± 257.42	1688.15 ± 354.21
Cl_tot_/*F* (L/h)			
Day 1	0.86 ± 0.24	0.83 ± 0.14	0.79 ± 0.61
Day 7	0.64 ± 0.17	0.66 ± 0.11	0.61 ± 0.13
kel (1/h)			
Day 1	0.0608 ± 0.0101	0.0686 ± 0.0089	0.0627 ± 0.0104
Day 7	0.0611 ± 0.0071	0.0666 ± 0.0043	0.0644 ± 0.0051
Vd/*F* (L)			
Day 1	13.93 ± 2.59	12.14 ± 0.82	12.84 ± 1.64
Day 7	10.39 ± 1.94	9.81 ± 1.27	9.52 ± 1.75
MRT_0-inf_ (h)			
Day 1	17.68 ± 3.02	15.74 ± 2.00	16.77 ± 3.38
Day 7	16.78 ± 2.23	14.88 ± 1.09	15.53 ± 1.63

Data are presented as mean ± SD

#### PK of dotinurad in the combination group

In the combination group, the mean plasma dotinurad concentrations were not affected by topiroxostat at all timepoint on day 1 compared with those of the overproduction group (Fig. 3). All PK parameters of dotinurad with/without coadministration of topiroxostat were also comparable (Table 2).

### Pharmacodynamics

#### Serum uric acid in the overproduction and underexcretion groups

**Fig. 4:**
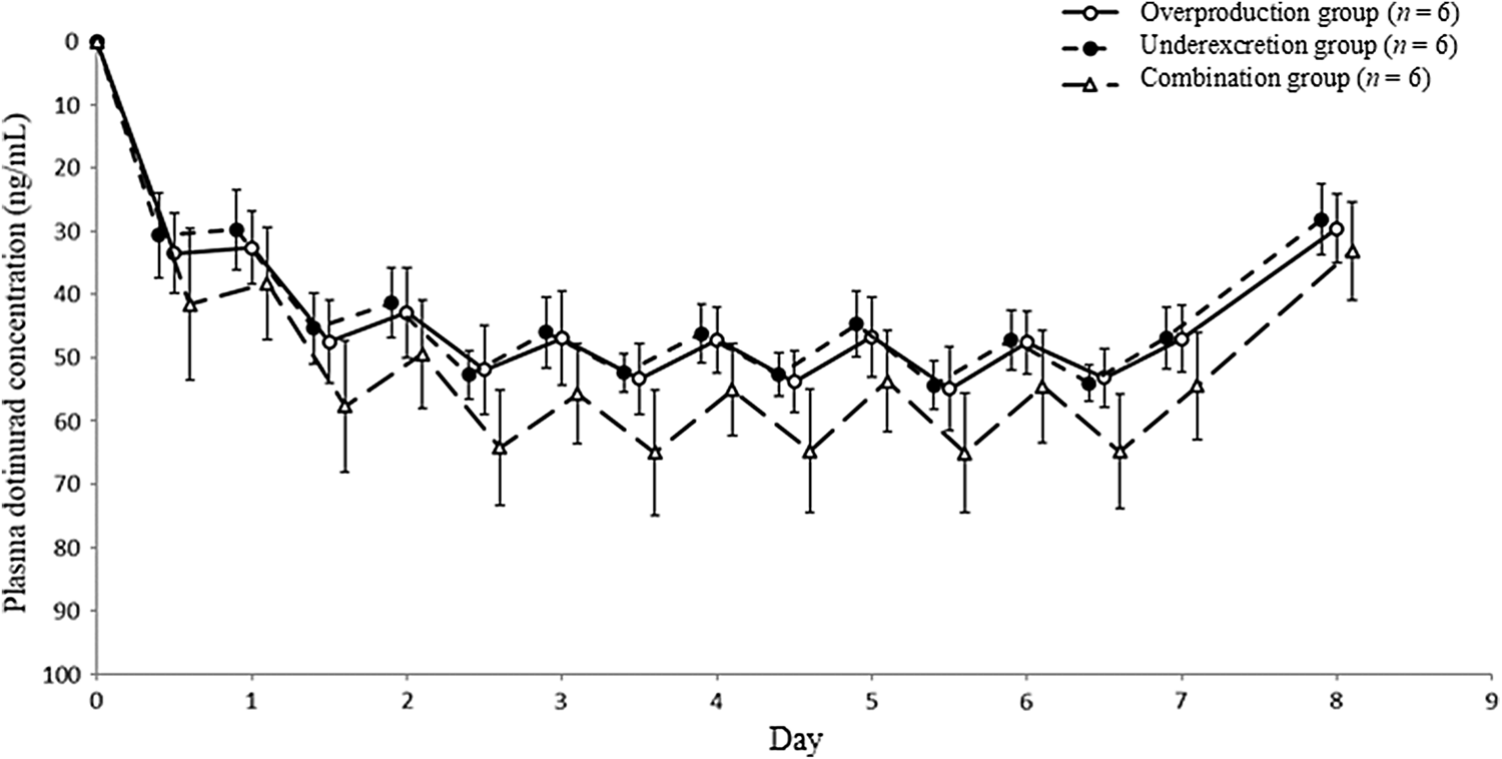
Percent change in serum uric acid level in the overproduction, underexcretion, and combination groups. Error bars indicates standard deviation

The percent change in serum uric acid level after the initial administration of dotinurad increased in a similar manner in both the overproduction group and the underexcretion group (Fig. 4). Additionally, the ΔEC_max_ and maximum reduction rates were comparable in both groups (Table 3). Regarding the ΔAUEC_0–24_ on days 1, 4, and 7, no significant differences were observed at all timepoints in both groups.

**Table 3:** PD parameters of serum uric acid in the overproduction, underexcretion, and combination groups

Parameters	Treatment groups			*P* value^a^
	Overproduction (*n* = 6)	Underexcretion (*n* = 6)	Combination (*n* = 6)	Underexcretion/combination
ΔEC_max_ (mg/dL)	− 4.60 ± 0.53	− 4.83 ± 0.35	− 5.88 ± 0.50	
ΔAUEC_0–24_ (mg∙h/dL)				
Day 1	52.90 ± 9.71	51.93 ± 7.38	71.72 ± 16.46	0.985/0.027*
Day 4	99.36 ± 11.20	105.69 ± 7.10	129.05 ± 12.91	0.501/< 0.001*
Day 7	99.73 ± 8.70	107.37 ± 9.38	127.98 ± 16.55	0.460/0.002*
Maximum reduction (%)	56.86 ± 6.80	55.75 ± 4.48	68.19 ± 7.70	

Data are presented as mean ± SD

*P < 0.05 (vs overproduction group)

^a^Dunnett multiple comparison test was applied to the underexcretion group and the combination group, setting the overproduction group as the control group

#### Urinary uric acid in the overproduction and underexcretion groups

**Fig. 5:**
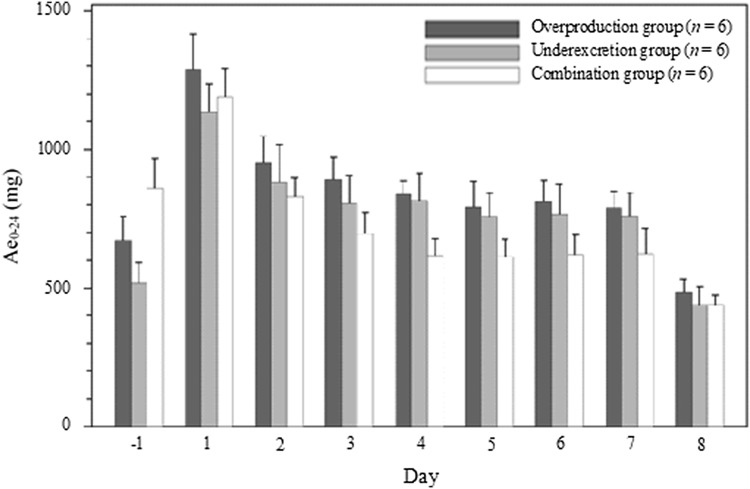
Change in the urinary uric acid excretion in the overproduction, underexcretion, and combination groups. Error bars indicates standard deviation. *Ae* amount of urate excreted in urine

In the overproduction and underexcretion groups, Ae_0–24_ fluctuated in a similar manner (Fig. 5, Table 4). Ae_0–24_ on day − 1 of both groups were 673.40 and 516.25 mg, and on day 1 increased to 1289.33 and 1132.30 mg, respectively. In contrast, on day 2, those decreased to 951.45 and 882.17 mg, respectively. From day 4, these fluctuations were comparable degree in both groups.

**Table 4:** PD parameters of urine uric acid levels in the overproduction, underexcretion, and combination groups

Parameters	Treatment groups			*P* value^a^
	Overproduction (*n* = 6)	Underexcretion (*n* = 6)	Combination (*n* = 6)	Underexcretion/Combination
Ae_0–24_ (mg)				
Day – 1	673.40 ± 85.52	516.25 ± 76.62	858.92 ± 108.63	–
Day 1	1289.33 ± 126.45	1132.30 ± 104.21	1187.77 ± 104.29	–
Day 2	951.45 ± 95.98	882.17 ± 132.88	829.33 ± 66.85	–
Day 3	890.43 ± 82.41	804.60 ± 101.73	695.25 ± 75.70	–
Day 4	838.15 ± 48.69	816.02 ± 98.22	616.40 ± 62.45	–
Day 5	792.85 ± 91.37	758.12 ± 84.21	612.55 ± 64.36	–
Day 6	810.43 ± 77.87	765.65 ± 110.89	619.17 ± 72.05	–
Day 7	790.30 ± 58.99	760.50 ± 82.88	621.88 ± 92.18	–
Day 8	483.35 ± 48.80	439.25 ± 65.28	438.77 ± 36.35	–
CL_R0–24_ (mL/min)				
Day – 1	5.77 ± 0.64	4.14 ± 0.68	6.96 ± 1.33	0.017*/0.079
Day 1	15.34 ± 2.21	12.50 ± 3.18	15.25 ± 4.93	0.361/0.999
Day 4	14.92 ± 2.03	13.33 ± 1.75	13.86 ± 3.80	0.500/0.721
Day 7	14.13 ± 2.23	12.60 ± 1.36	13.60 ± 3.53	0.490/0.909
FE_0–24_ (%)				
Day – 1	4.77 ± 0.89	3.57 ± 0.64	4.93 ± 1.08	0.060/0.939
Day 4	12.29 ± 2.04	11.04 ± 2.26	10.35 ± 2.10	0.511/0.232
Day 7	11.76 ± 1.68	10.26 ± 1.51	10.25 ± 2.33	0.308/0.305

Data are presented as the mean ± SD

*P < 0.05 (vs overproduction group)

^a^Dunnett multiple comparison test was applied to the underexcretion group and the combination group, setting the overproduction group as the control group

Comparison of uric acid clearance (CL_R0–24_) on days 1, 4, and 7 in both groups, no significant differences were observed at all timepoints. Furthermore, regarding FE_0–24_ on days 4 and 7, no significant differences were observed at all timepoints.

#### Serum uric acid in the combination group

In the combination group, the percent change in serum uric acid level increased at all timepoints compared to those of the overproduction group (Fig. 4). Furthermore, the ΔEC_max_ and maximum reduction rate were higher than those of the overproduction group. When comparing the ΔAUEC_0–24_ of the combination group to those of the overproduction group, significant differences were observed at all timepoints (Table 3).

#### Urinary uric acid in the combination group

The fluctuation of Ae_0–24_ in the combination group was similar to those in the overproduction group (Fig. 5, Table 4). At all timepoints, Ae_0–24_ in the combination group were lower than those in the overproduction group.

Regarding uric acid clearance (CL_R0–24_) and FE_0–24_, no significant differences were observed between the overproduction group and the combination group, at all timepoints.

### Safety

The AE incidence rates were 33.3% (two patients, eight events) in the underexcretion group, 16.7% (one patient, one events) in the combination group, and none in the overproduction group (Table 5). An ADR was observed in one patient (alanine aminotransferase increased) in the combination group. The severity of the ADR was mild, and the patient recovered without treatment. No serious adverse event was observed in any group.

**Table 5:** Summary of adverse events

Adverse events	Treatment groups		
	Overproduction	Underexcretion	Combination
	(*n* = 6)	(*n* = 6) (%)	(*n* = 6) (%)
All	0 (0.0%)	2 (33.3)	1 (16.7)
Nervous system disorders	0 (0%)	1 (16.7%)	0 (0%)
Headache	0 (0.0%)	1 (16.7)	0 (0.0)
Eye disorders	0 (0%)	1 (16.7%)	0 (0%)
Eye pain	0 (0.0%)	1 (16.7)	0 (0.0)
Respiratory, thoracic and mediastinal disorders	0 (0%)	2 (33.3%)	0 (0%)
Oropharyngeal swelling	0 (0.0%)	1 (16.7)	0 (0.0)
Oropharyngeal pain	0 (0.0%)	1 (16.7)	0 (0.0)
Gastrointestinal disorders	0 (0%)	1 (16.7%)	0 (0%)
Abdominal distension	0 (0.0%)	1 (16.7)	0 (0.0)
Abdominal pain	0 (0.0%)	1 (16.7)	0 (0.0)
Musculoskeletal and connective tissue disorders	0 (0%)	1 (16.7%)	0 (0%)
Neck pain	0 (0.0%)	1 (16.7)	0 (0.0)
Investigations	0 (0%)	0 (0%)	1 (16.7%)
Alanine aminotransferase increased	0 (0.0%)	0 (0.0)	1 (16.7)

Incidence (%) = number of patients/number of analyzed patients × 100

## Discussion

### Difference of hyperuricemic types

This study showed no differences in the PK of dotinurad between the overproduction and underexcretion groups. Regarding PD, serum uric acid lowering effects were comparable and the time-course of Ae_0–24_ was similar in both groups. Moreover, when dotinurad was administered continuously, the Ae_0–24_ in both groups indicated comparable levels. There were no clinically meaningful differences in safety between patients with these hyperuricemic types.

We considered that it was necessary to investigate what degree urinary uric acid excretion changes when dotinurad is administered repeatedly in “overproduction type” patients, as the drug promotes uric acid excretion into urine. From our study results, “overproduction type” patients in whom urinary uric acid excretion increases continuously were not observed, and the urinary uric acid excretion temporarily increased but later decreased. This was similar to fluctuations of urinary uric acid excretion found in “underexcretion type” patients. Furthermore, continuous administration of dotinurad results in the comparable amount of urinary uric acid excretion in both type patients. Therefore, we conjectured that the risk of urinary calculus with dotinurad may not be substantially different between these two types.

### Coadministration of XOI (topiroxostat)

In “overproduction type” patients, we found that concomitant administration of dotinurad and topiroxostat showed an add-on serum uric acid lowering effect and suppression of urinary uric acid excretion, compared to the administration of dotinurad monotherapy. We hypothesize that the concomitant use of topiroxostat reduced the amount of uric acid production in the body, thereby suppressing urinary uric acid excretion. No clinically meaningful differences in safety were observed between dotinurad monotherapy and concomitant therapy with topiroxostat.

In this study, with patient lifestyle controlled under hospitalization, all patients classified as the “overproduction type” at screening had changed to other hyperuricemic type by day − 1, even though dotinurad had not been administered. Most of these patients changed to “underexcretion type” (Table 6). Conversely, changes in hyperuricemic type were rarely observed in “underexcretion type” patients (Table 7). Therefore, most of the patients classified as “overproduction type” may have fallen into temporary overproduction condition of uric acid due to the effects of environmental factors (e.g. excessive intake of purines or calories, or alcohol consumption). We can therefore speculate that only a few “overproduction type” patients are classified as such due to intrinsic factors, and most are apparent “overproduction type” due to environmental factors associated with their individual lifestyle.

**Table 6:** Change in hyperuricemic type in inpatients with overproduction

	Hyperuricemic type (Screening vs day – 1)		Total
	Changed	Unchanged	
With hospitalization (original protocol)^a^	16	0	16
Without hospitalization (amendment)^b^	15	10	25
Total	31	10	41

^a^Patients who were hospitalized during the run-in period and had restricted daily living including eating and drinking

^b^Patients who were not hospitalized during the run-in period and maintained their normal activities of daily living

**Table 7:** Change in hyperuricemic type in inpatients with underexcretion

	Hyperuricemic type (Screening vs day – 1)		Total
	Changed	Unchanged	
With hospitalization (original protocol)^a^	1	14	15
Without hospitalization (amendment)^b^	6	34	40
Total	7	48	55

^a^Patients who were hospitalized during the run-in period and had restricted daily living including eating and drinking

^b^Patients who were not hospitalized during the run-in period and maintained normal activities of daily living

From the above, concomitant therapy with dotinurad and XOI appears to be a reasonable treatment. In Japan, although the primary cause for hyperuricemia is considered to be decreased uric acid excretion, the findings obtained in this study suggest that many “underexcretion type” patients exhibit temporary overproduction characteristics due to lifestyle-associated environmental factors. In this study, most of the patients classified as “overproduction type” changed to “underexcretion type” when lifestyle was controlled. In considering the disposition of uric acid in many Japanese people with hyperuricemia, suppressing uric acid production while simultaneously promoting uric acid excretion in urine may be a reasonable treatment method.

This was the first study to administer dotinurad to “overproduction type” patients; thus, it was conducted as a 7-day, multiple-dose study under conditions of hospitalization where meal and lifestyle environments were strictly controlled. In the future, however, long-term clinical studies must be conducted in outpatients to generalize the results obtained in this study.

## Electronic supplementary material

Below is the link to the electronic supplementary material.

Supplementary material 1 (DOCX 1206 kb)
