# Supplementary material for: The effect for hyperuricemia inpatient of uric acid overproduction type or in combination with topiroxostat on the pharmacokinetics, pharmacodynamics and safety of dotinurad, a selective urate reabsorption inhibitor
Source: Clin Exp Nephrol. 2019 Nov 16;24(Suppl 1):92–102. doi: 10.1007/s10157-019-01817-3 (PMC7066310; doi:10.1007/s10157-019-01817-3)
Supplement: Supplementary file 1 — Supplementary material 1 (DOCX 1206 kb) [file 10157_2019_1817_MOESM1_ESM.docx]

**Supplement 1** Change in plasma concentration of dotinurad on Day 1 in the overproduction, underexcretion, combination group, and reference groups

Error bars indicates standard deviation.

※：There were 5 patients after Day 4 in the reference group.

Overproduction group (*n* = 6)

Underexcretion group (*n* = 6)

Combination group (*n* = 6)

Reference group (*n* = 6)

※

**Supplement 2** Percent change in serum uric acid level in the overproduction, underexcretion, combination group, and reference groups

Error bars indicates standard deviation.

※：There were 5 patients after Day 4 in the reference group.

※

Overproduction group (*n* = 6)

Underexcretion group (*n* = 6)

Combination group (*n* = 6)

Reference group (*n* = 6)

**Supplement 3** Change in the urinary uric acid excretion in the overproduction, underexcretion, combination group, and reference groups

Day

Error bars indicates standard deviation.

※：There were 5 patients after Day 4 in the reference group.

*Ae* amount of urate excreted in urine.


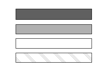

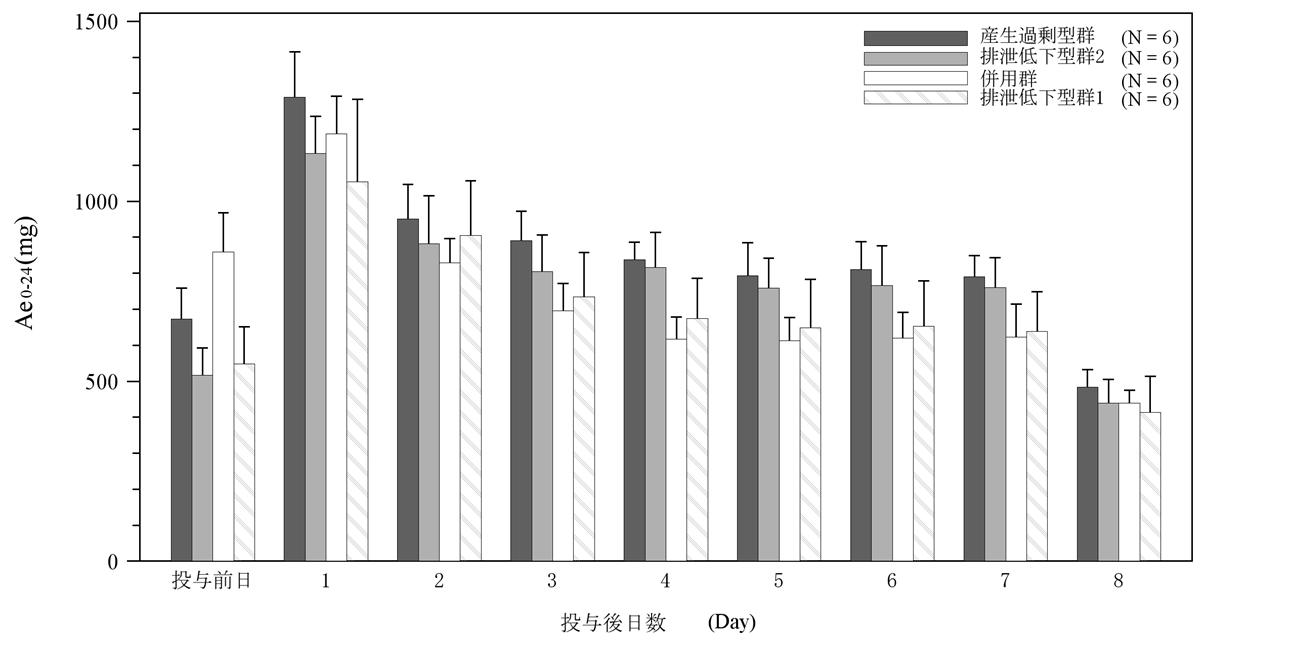


※

Day

Overproduction group (*n* = 6)

Underexcretion group (*n* = 6)

Combination group (*n* = 6)

Reference group (*n* = 6)

Ae_0-24_ (mg)

**Supplement 4** Summary of the baseline characteristics

|  | Treatment groups | | | |
| --- | --- | --- | --- | --- |
| Category | Overproduction  (*n* = 6) | Underexcretion  (*n* = 6) | Combination  (*n* = 6) | Reference  (*n* = 6) |
|  |  |  |  |  |
| Age (year) | 63.0 ± 9.7 | 52.8 ± 8.2 | 50.3 ± 6.0 | 54.7 ± 12.8 |
| Height (cm) | 170.85 ± 4.73 | 164.35 ± 2.54 | 169.32 ± 3.68 | 164.83 ± 4.86 |
| Weight (kg) | 72.62 ± 9.85 | 69.40 ± 6.54 | 77.67 ± 7.62 | 65.20 ± 7.61 |
| BMI (kg/m^2^) | 24.92 ± 3.68 | 25.75 ± 2.98 | 27.05 ± 2.01 | 23.98 ± 2.45 |
| eGFR (mL/min/1.73 m^2^) | 74.0 ± 14.1 | 73.7 ± 8.4 | 80.8 ± 4.4 | 76.0 ± 8.2 |
| Serum uric acid level (mg/dL) | 7.68 ± 0.47 | 8.30 ± 0.93 | 8.32 ± 0.50 | 7.82 ± 0.44 |

BMI, body mass index.

eGFR (mL/min/1.73 m^2^) = 194 × Serum creatinine^-1.094^ × Age^-0.287^.

Values are presented as the mean ± SD.

**Supplement 5** PK parameters of dotinurad in plasma and urine on Day 1 and 7 in the overproduction, underexcretion, combination group, and reference groups

|  |  | Treatment groups | | | |
| --- | --- | --- | --- | --- | --- |
| Parameters |  | Overproduction  (*n* = 6) | Underexcretion  (*n* = 6) | Combination  (*n* = 6) | Reference  (*n* = 6) |
|  |  |  |  |  |  |
| C_max_ (ng/mL) | Day 1 | 78.68 ± 14.92 | 83.80 ± 9.77 | 83.28 ± 10.22 | 103.98 ± 17.16 |
|  | Day 7 | 102.90 ± 21.43 | 101.20 ± 18.28 | 108.33 ± 15.49 | 127.48 ± 24.96 |
|  |  |  |  |  |  |
| T_max_ (hr) | Day 1 | 3.50 ± 0.55 | 4.00 ± 0.00 | 3.17 ± 0.41 | 3.67 ± 0.52 |
|  | Day 7 | 3.67 ± 0.52 | 3.17 ± 0.98 | 3.67 ± 0.52 | 4.00 ± 0.00 |
|  |  |  |  |  |  |
| T_1/2_ (hr) | Day 1 | 11.67 ± 1.92 | 10.24 ± 1.32 | 11.37 ± 2.23 | 11.73 ± 1.47 |
|  | Day 7 | 11.49 ± 1.49 | 10.44 ± 0.66 | 10.83 ± 0.96 | 11.86 ± 0.93 |
|  |  |  |  |  |  |
| AUC_0-inf_  (ng･hr/mL) | Day 1 | 1277.29 ± 490.68 | 1224.51 ± 197.78 | 1274.93 ± 155.18 | 1592.06 ± 341.17 |
|  | Day 7 | 1688.49 ± 633.70 | 1561.90 ± 257.42 | 1688.15 ± 354.21 | 2164.67 ± 414.50 |
|  |  |  |  |  |  |
| Cl_tot_/F (L/hr) | Day 1 | 0.86 ± 0.24 | 0.83 ± 0.14 | 0.79 ± 0.61 | 0.65 ± 0.14 |
|  | Day 7 | 0.64 ± 0.17 | 0.66 ± 0.11 | 0.61 ± 0.13 | 0.47 ± 0.08 |
|  |  |  |  |  |  |
| kel (1/hr) | Day 1 | 0.0608 ± 0.0101 | 0.0686 ± 0.0089 | 0.0627 ± 0.0104 | 0.0599 ± 0.0078 |
|  | Day 7 | 0.0611 ± 0.0071 | 0.0666 ± 0.0043 | 0.0644 ± 0.0051 | 0.0587 ± 0.0047 |
|  |  |  |  |  |  |
| Vd/F (L) | Day 1 | 13.93 ± 2.59 | 12.14 ± 0.82 | 12.84 ± 1.64 | 10.84 ± 1.40 |
|  | Day 7 | 10.39 ± 1.94 | 9.81 ± 1.27 | 9.52 ± 1.75 | 8.06 ± 1.12 |
|  |  |  |  |  |  |
| MRT_0-inf_ (hr) | Day 1 | 17.68 ± 3.02 | 15.74 ± 2.00 | 16.77 ± 3.38 | 18.23 ± 1.90 |
|  | Day 7 | 16.78 ± 2.23 | 14.88 ± 1.09 | 15.53 ± 1.63 | 17.22 ± 1.17 |

Data are presented as mean ± SD.

**Supplement 6** PD parameters of serum uric acid in the overproduction, underexcretion, combination group, and reference groups

|  |  | Treatment groups | | | |
| --- | --- | --- | --- | --- | --- |
| Parameters |  | Overproduction  (*n* = 6) | Underexcretion  (*n* = 6) | Combination  (*n* = 6) | Reference  (*n* = 6) |
|  |  |  |  |  |  |
| ΔEC_max_ (mg/dL) |  | −4.60 ± 0.53 | −4.83 ± 0.35 | −5.88 ± 0.50 | −4.75 ± 0.54 |
|  |  |  |  |  |  |
|  | Day 1 | 52.90 ± 9.71 | 51.93 ± 7.38 | 71.72 ± 16.46 | 50.62 ± 8.43 |
| ΔAUEC_0-24_ (mg・hr/dL) | Day 4 | 99.36 ± 11.20 | 105.69 ± 7.10 | 129.05 ± 12.91 | 106.18 ± 9.92 |
|  | Day 7 | 99.73 ± 8.70 | 107.37 ± 9.38 | 127.98 ± 16.55 | 108.39 ± 9.91 |
|  |  |  |  |  |  |
| Maximum reduction (%) |  | 56.86 ± 6.80 | 55.75 ± 4.48 | 68.19 ± 7.70 | 58.34 ± 4.31 |

*: *P* < 0.05 (vs overproduction group).

Data are presented as mean ± SD.

a: Dunnett multiple comparison test was applied to the underexcretion group and the combination group, setting the overproduction group as the control group.

**Supplement 7** PD parameters of urine uric acid levels in the overproduction, underexcretion, combination group, and reference groups

|  |  | Treatment groups | | | |
| --- | --- | --- | --- | --- | --- |
| Parameters |  | Overproduction  (*n* = 6) | Underexcretion  (*n* = 6) | Combination  (*n* = 6) | Reference  (*n* = 6) |
|  |  |  |  |  |  |
|  | Day −1 | 673.40 ± 85.52 | 516.25 ± 76.62 | 858.92 ± 108.63 | 547.45 ± 103.09 |
|  | Day 1 | 1289.33 ± 126.45 | 1132.30 ± 104.21 | 1187.77 ± 104.29 | 1054.00 ± 228.88 |
|  | Day 2 | 951.45 ± 95.98 | 882.17 ± 132.88 | 829.33 ± 66.85 | 904.42 ± 151.84 |
|  | Day 3 | 890.43 ± 82.41 | 804.60 ± 101.73 | 695.25 ± 75.70 | 733.67 ± 123.44 |
| Ae_0-24_ (mg) | Day 4 | 838.15 ± 48.69 | 816.02 ± 98.22 | 616.40 ± 62.45 | 674.38 ± 111.70 |
|  | Day 5 | 792.85 ± 91.37 | 758.12 ± 84.21 | 612.55 ± 64.36 | 648.14 ± 135.57 |
|  | Day 6 | 810.43 ± 77.87 | 765.65 ± 110.89 | 619.17 ± 72.05 | 652.22 ± 125.98 |
|  | Day 7 | 790.30 ± 58.99 | 760.50 ± 82.88 | 621.88 ± 92.18 | 638.16 ± 110.98 |
|  | Day 8 | 483.35 ± 48.80 | 439.25 ± 65.28 | 438.77 ± 36.35 | 413.06 ± 99.97 |
|  |  |  |  |  |  |
|  | Day −1 | 5.77 ± 0.64 | 4.14 ± 0.68 | 6.96 ± 1.33 | 4.65 ± 0.58 |
| CL_R0-24_ (mL/min) | Day 1 | 15.34 ± 2.21 | 12.50 ± 3.18 | 15.25 ± 4.93 | 12.13 ± 2.14 |
|  | Day 4 | 14.92 ± 2.03 | 13.33 ± 1.75 | 13.86 ± 3.80 | 12.33 ± 1.08 |
|  | Day 7 | 14.13 ± 2.23 | 12.60 ± 1.36 | 13.60 ± 3.53 | 11.95 ± 1.40 |
|  |  |  |  |  |  |
|  | Day −1 | 4.77 ± 0.89 | 3.57 ± 0.64 | 4.93 ± 1.08 | 3.71 ± 0.43 |
| FE_0-24_ (%) | Day 4 | 12.29 ± 2.04 | 11.04 ± 2.26 | 10.35 ± 2.10 | 9.88 ± 1.73 |
|  | Day 7 | 11.76 ± 1.68 | 10.26 ± 1.51 | 10.25 ± 2.33 | 9.58 ± 1.54 |

*: *P* < 0.05 (vs overproduction group).

Data are presented as the mean ± SD.
a: Dunnett multiple comparison test was applied to the underexcretion group and the combination group, setting the overproduction group as the control group.

**Supplement 8** Summary of adverse events

| Adverse events | | Treatment groups | | | |
| --- | --- | --- | --- | --- | --- |
|  |  | Overproduction  (*n* = 6) | Underexcretion  (*n* = 6) | Combination  (*n* = 6) | Reference  (*n* = 6) |
|  |  |  |  |  |  |
| All | | 0 (0.0%) | 2 (33.3%) | 1 (16.7%) | 1 (16.7%) |
| Nervous system disorders | | 0 (0%) | 1 (16.7%) | 0 (0%) |  |
|  | Headache | 0 (0.0%) | 1 (16.7%) | 0 (0.0%) | 0 (0.0%) |
| Eye disorders | | 0 (0%) | 1 (16.7%) | 0 (0%) |  |
|  | Eye pain | 0 (0.0%) | 1 (16.7%) | 0 (0.0%) | 0 (0.0%) |
| Respiratory, thoracic and mediastinal disorders | | 0 (0%) | 2 (33.3%) | 0 (0%) |  |
|  | Oropharyngeal swelling | 0 (0.0%) | 1 (16.7%) | 0 (0.0%) | 0 (0.0%) |
|  | Oropharyngeal pain | 0 (0.0%) | 1 (16.7%) | 0 (0.0%) | 0 (0.0%) |
| Gastrointestinal disorders | | 0 (0%) | 1 (16.7%) | 0 (0%) |  |
|  | Abdominal distension | 0 (0.0%) | 1 (16.7%) | 0 (0.0%) | 0 (0.0%) |
|  | Abdominal pain | 0 (0.0%) | 1 (16.7%) | 0 (0.0%) | 0 (0.0%) |
| Musculoskeletal and connective tissue disorders | | 0 (0%) | 1 (16.7%) | 0 (0%) |  |
|  | Neck pain | 0 (0.0%) | 1 (16.7%) | 0 (0.0%) | 0 (0.0%) |
|  | Pain in extremity | 0 (0.0%) | 0 (0.0%) | 0 (0.0%) | 1 (16.7%) |
| Investigations | | 0 (0%) | 0 (0%) | 1 (16.7%) |  |
|  | Alanine aminotransferase increased | 0 (0.0%) | 0 (0.0%) | 1 (16.7%) | 1 (16.7%) |
|  | Blood creatine phosphokinase increased | 0 (0.0%) | 0 (0.0%) | 0 (0.0%) | 1 (16.7%) |

Incidence (%) = number of patients/number of analyzed patients × 100.
